# Delivering person-centered care with an electronic health record

**DOI:** 10.1186/s12911-019-0897-6

**Published:** 2019-08-22

**Authors:** Victoria Stanhope, Elizabeth B. Matthews

**Affiliations:** 1 Silver School of Social Work, New York University, 1 Washington Square North, New York, NY 10003 USA; 2 Graduate School of Service, Fordham University, 113 West 60th Street, New York, NY 10023 USA

**Keywords:** Person-centered care, Electronic health records, Health information technology, Mental health services

## Abstract

**Background:**

Electronic health records are now widely adopted in medical and behavioral health settings. While they have the potential to improve the quality of care, the research findings on their impact on clinical practice and outcomes have been mixed. This study explores how the electronic health record and its stage of development influenced the implementation of person-centered care planning in community mental health clinics.

**Methods:**

The study was set in five community mental health clinics which utilized an EHR and had been trained in person-centered care planning. Using an objective quantitative measure of fidelity, the study examined fidelity to PCCP across time and by stage of EHR development. Data from focus groups, interviews with clinic leaders and consultant reports was analyzed to explore the process of implementation and the role of the electronic health record.

**Results:**

All clinics demonstrated an overall increase in PCCP fidelity at the conclusion of the study period but there were significant differences in PCCP fidelity among clinics with EHRs in different stages of development. Electronic health records emerged as a significant implementation factor in the qualitative data with clinics being unable to individualize service plans and encountering technical difficulties. Barriers to person-centered care included drop-down boxes and pre-determined outcomes. Clinic responses included customizing their record or developing workarounds.

**Conclusions:**

The study demonstrated the need to align the electronic health record with a person-centered approach which includes individualizing information and orienting service plans to personal life goals. The ability of clinics to be able to customize their records and balance the need for unique and aggregate information in the record is critical to improve both the provider experience and the quality of care.

**Trial registration:**

Clinicaltrials.gov, NCT02299492, registered on November 24, 2014.

## Contributions to the literature

- Research on the impact of the electronic health records on clinical practice and outcomes has been mixed. The findings from this mixed methods study provide insight into the “black box” of how the EHR reflects and influences clinical practice.
- In this study, we found both implementation barriers with regard to usability of the EHR with regards to service planning and the ability to individualize client information, which is a key aspect of person-centered care.
- These findings demonstrate the importance of aligning an electronic health record with person-centered care and being able to customize the EHR in response to implementation barriers.

## Introduction

Electronic health records (EHRs) have great potential to improve the quality of care by promoting effectiveness, efficiency, timely, patient-centered care, safety and equity [1, 2]. This digital technology has become a key strategy in health care reform efforts with the EHR adoption by health care organizations being incentivized by policies such as the Affordable Care Act and the Health Information Technology for Economic and Clinical Health Act. Research on EHRs has focused primarily on outcomes related to efficiency and effectiveness with positive findings related to accessing health care, reducing costs, monitoring population health care and improving disease specific outcomes [3, 4]. However, there is also evidence showing how EHRs can be a detriment to health care delivery due to disruptions in workflow, provider resistance and lack of compatibility with existing health practices [5]. This has led to calls for research examining the “black box” of EHR adoption to fully understand how adoption and sustainment influences clinical practice.

While the majority of the research on EHRs has been in medical settings, this study focuses on how they influence the delivery of person-centered care in behavioral health settings. Although slower to adopt than medical settings, a survey by the National Council for Behavioral Health in 2012 [6] found that 56% of behavioral organizations used some form of EHR system and an additional 26% of agencies stated were planning to implement an electronic system in the near future. The predominant impetus for the adoption of EHRs within behavioral health has been large-scale efforts to integrate care. Driven by the poor health outcomes among people with severe mental illnesses, there is now widespread integration of primary care, mental health and substance abuse to ensure that people are treated holistically [7, 8]. The electronic health record is the bedrock of these care coordination efforts, serving as the primary mechanism for facilitating communication among providers both within fully integrated settings and across networked providers [9]. One of the most prevalent models within behavioral health has been the Health Home model which requires the adoption of an EHR system or utilization of HIT in some form [10].

Integrated care is by definition person-centered care, a way of delivering services that pays attention to the whole person. Part of a larger movement within health care, person-centered care is broadly defined as care that is “respectful of and responsive to individual patient preferences, needs, and values, and ensures that patient values guide all clinical decisions” [2]. Within integrated health care models, the emphasis has been on promoting the activated patient, who is empowered to be fully involved in making decisions and shaping their own care. Person-centered care is also a key component of the mental health recovery movement, a paradigm shift in service orientation that has been driving mental health reform since it became a federal priority in the early 2000s [11]. Recovery challenges the medical model by reorienting care from symptom reduction to focusing care on supporting people on their own unique recovery journey [12]. This entails providers working towards treatment goals that look beyond symptom management to helping people live a meaningful life in the community.

An emerging recovery-oriented practice is person-centered care planning, which uses the service planning process to develop and implement an action plan to assist the person in achieving his or her unique, personal life goals [13, 14]. Providers complete a service plan with clients on entry into a program and then update the plan every 3 to 6 months. This plan addresses the specific mental health and/or substance use barriers interfering with the person’s goal achievement using both professional services and natural supports. Throughout the care planning process, providers elicit and empathize with their clients’ subjective experiences, regard clients holistically as people rather than as patients, and help people to articulate their personal recovery goals. Providers then reflect this process in the service plan wherever possible using the service user’s own words. Skills include reframing symptoms and impairments as barriers to goal attainment; reframing the use of medications as tools for overcoming these barriers and moving ahead in one’s life; instilling hope and identifying short-term, realistic, and measurable objectives. Overall, this approach is framed by a perspective that focuses on the application of clients’ strengths rather than the treatment of their deficits [14].

Person-centered care is therefore reflected, communicated to others, and promoted by the service plan, which embodies both the spirit and specifics of treatment. As the act of service planning transitions over to a digital medium, this process is increasingly embedded within the electronic health record. In this regard, the functioning of the EHR and the delivery person-centered practices are necessarily shaping and shaped by one another. In a study examining the value of the EHR in the delivery of mental health care in Ontario, researchers describe how EHR driven initiatives did promote person-centered care but stressed that careful planning and consideration is needed to optimally integrate clinical decision support as EHR functions become more complex [1].

Electronic health records have many purposes, including documentation for reimbursement and tracking population health data, but must also have the ability to capture individualized data that can be communicated among multiple providers and inform ongoing care. The shift from paper to electronic documentation has obvious benefits in terms of ease of access, less error, and more legibility, but how it impacts the quality of documentation is less clear [15]. Research in nursing has found that person-centered care is often being practiced among providers but not documented [16]. This finding reflects a common provider perception that documentation is “busywork” without clinical value and detracts from their therapeutic interactions with service users.

The reason for a lack of person-centeredness in documentation can be that the EHR itself is formatted in such a way that precludes providers capturing the individual person in terms of key biographical details [17, 18]. In mental health settings, where a client’s narrative is a central part to the treatment plan, the need to fit these narratives to the constraints of the fields of entry is part of the service planning process. Triplett [19] describes how open-ended questions in a clinical interview can become closed ended questions when they are driven by drop down boxes and computerized decision trees. The role of the template, while also a factor in service planning on paper, takes on an added significance as the information entered has multiple purposes and is made available to different providers.

Some research suggests that the impact of EHRs on clinical quality may change over time, particularly as a function of the organization’s stage of EHR implementation. For example, a systematic review examining the impact of EHR systems on clinical documentation found that, while providers spent more time on documentation immediately following EHR adoption, this effect may reverse over time, as both organizations and individual providers become more familiar with the system [20]. Similarly, a study exploring the role of EHRs in integrating primary care and behavioral health found that optimizing the system was both an iterative and long-term process [3]. Additional research has found that the unique clinical benefit of these systems can take between 2 and 4 years to be fully realized [21, 22]. Consequently, considering the stage of EHR adoption, including how long a particular system has been in use, may be a critical component in understanding its differential impact on care.

In an extensive review of how patient-centered care can be facilitated by health information technology, Finkelstein [23] found that “depersonalization” was one of the barriers cited in multiple studies and called for more research on the how health information technology impacts person-centered care. This mixed methods study explores how the electronic health record and its stage of development influenced the implementation of person-centered care planning in community mental health clinics.

## Methods

Using a convergent parallel mixed methods design, the study utilized quantitative data to examine differences in fidelity to PCCP among clinics according to EHR development stage and qualitative data to explore the role of the electronic health record in PCCP fidelity from the provider perspective. This study was part of a larger multi-site randomized controlled trial of person-centered care planning (Authors, 2013). The study received approval from a university Institutional Review Board.

### Study setting

The study was set within five mental health clinics across two states that had been randomized to the PCCP condition in the randomized controlled trial. These clinics provided a range of services including outpatient therapy, crisis intervention, medication management, case management, residential programs, community support programs, and rehabilitation services. Within these clinics, leadership, supervisors, and direct care staff participated in the study. The clinics were selected for this study on the basis that they were in the experimental condition and were actively using an EHR. Each of the clinics received training in PCCP, which consisted of a two-day in-person training session at the agency followed by monthly technical assistance (TA) calls facilitated by external PCCP consultants over a 12-month period. Each clinic had two TA calls a month. One call was with supervisors to reinforce their translation of the practice to their direct care provider teams and one call was with a clinic team which provided a service plan for feedback from the consultants.

### Qualitative methods

Qualitative data was collected from 11 focus groups, 12 interviews and 24 consultant reports from each site. The focus groups and leadership interviews asked questions about the implementation process of PCCP. One supervisor focus group and one direct care focus group was conducted at each of the five clinics except for one clinic in which two direct care focus groups were conducted due to the large number of direct care staff participating. Twelve in-depth interviews were conducted with participant executive leaders of the clinics. The third data source was consultant reports that were completed after each of the bi-monthly technical assistance calls. Consultants were external experts who conducted the trainings and led the technical assistance calls. Using a template, the consultant completed a monthly narrative report on progress made in the implementation of PCCP, including barriers, strategies to address barriers, and leadership involvement for each clinic.

At the conclusion of the 12-month intervention period for each site, focus groups and leadership interviews were conducted at each experimental site to explore barriers and facilitators to implementation. Topical domains addressed during the focus groups included: perspectives on PCCP, experiences with implementing PCCP and training staff, including the role of the electronic health record. Each of the focus groups was conducted by two masters-level interviewers with experience working in community mental health clinics and training in the PCCP intervention. Focus groups lasted approximately 60 min. All participants received $20 compensation for their time. The qualitative sample included 31 clinical supervisors participating across the five supervisor focus groups and 52 direct care staff participating in the six direct care focus groups. Focus groups consisted of three to twelve participants each. In-depth interviews were completed individually with each of the 12 executive leadership participants.

Focus groups and interviews were digitally recorded and transcribed verbatim with names and identifying information removed. Transcripts were then entered into *Atlas.ti* for data management and analysis. Analysis of all qualitative data was conducted by three researchers, who examined the five cases both within developmental categories and across these categories to discern similarities in experiences. Data was analyzed within each agency and then compared to other agencies within that category. Any inconsistencies in coding were resolved through consensus. In addition, consultant reports were analyzed by two researchers using inductive, thematic analysis to capture commonalities both between and within sites. Data was organized using memos, which provide a structured summary of key experiences and themes [24]. Memoing is an approach often associated with grounded theory [25] but has become a commonly employed tool across qualitative methods as a mechanism to draw out core meanings within complex data and facilitate team debriefing [24]. To ensure trustworthiness throughout the coding process, the researchers engaged in strategies to ensure rigor, including weekly team debriefings and the use of an audit trail [26].

### Quantitative methods

A chart review of service plans completed by study participants was conducted by researchers trained in the PCCP intervention. At each of the 5 clinics, service plans were sampled from plans completed by providers that had received PCCP training. Twenty service plans were randomly selected at each of the timepoints (baseline, 12 months and 18 months) generating a total sample of 300 service plans. Each service plan reviewed was from a unique client.

Clinics were categorized according to the following development stages of their electronic health record: 1) electronic health record established and no change planned (*N* = 1); 2) electronic health record in transition with changes being actively implemented (*N* = 2); and 3) electronic health record established but further changes planned (*N* = 2). Clinic 1 was categorized as ‘no change’. This site had an existing EHR that remained stable throughout the PCCP implementation process, with no major transitions related to their system. Clinics 2 and 3 were categorized as ‘changing EHR’, as both were in the process of rolling out a new system at the baseline study period. Clinic 2 was in the midst of a phased roll-out of a new EHR system, while Clinic 3 had just implemented a new EHR across all programs. Finally, Clinics 4 and 5 were categorized as ‘planning change’. These sites both maintained an existing electronic system but were actively planning to transition to a new EHR platform during the implementation process. Clinic 4 was planning to upgrade from a basic computerized system to a commercial EHR, and Clinic 5 was part of a larger health care network that was collectively transitioning from one commercial system to another.

PCCP fidelity was measured by an objective measure, the PCCP Assessment Measure (PCCP-AM), that was applied to the service plans selected for chart review. This 13-item measure was developed by experts based on the PCCP manual [14]. The items assessed person-centeredness and technical proficiency across the key service planning domains including assessment, narrative summary, objectives, goals and interventions. The items were rated from 1 to 4 indicating level of competency: 1 indicated “needs improvement”; 2 indicated “approaching standard”; 3 indicated “meets standard”; 4 indicated “exceed standard.” For the purposes of the study, the scores were dichotomized by recoding items which scored 1 or 2 into 0 (not competent) and items which scored 3 or 4 into 1 (competent). Scores for each service plan ranged from 1 to 13 with higher scores indicating greater person-centeredness. For the parent study, 12 service plans were co-coded by two raters at each of the 14 research sites, which yielded an inter-rater reliability of 80%. The PCCP-AM had a Cronbach’s Alpha of .7 when calculated across all the service plans reviewed in the parent study (*N* = 798).

Univariate statistics were used to report PCCP fidelity at baseline, 12 months and 18 months. At each of the three time points, an ANOVA was run to test for differences in PCCP implementation between sites by the three stages of EHR adoption: planning a change, changing, or no change.

## Results

### Qualitative findings

There were 95 providers participating in the focus groups and interviews. The majority were female (*N* = 75, 80.6%), white (*N* = 64, 68.8%), and non-Hispanic (*N* = 88, 94.6%). The average age of providers was 45.15 (SD = 11.92). Within the sample, 36 (38.7%) had a bachelor’s degree and 51 (54.8%) had a master’s degree. Disciplines included social work, psychology and counseling. Years working at the agency ranged from less than one year to 37 years (M = 9.00, SD = 8.06).

In the qualitative findings, the EHR emerged as one of the most salient factors in PCCP fidelity. While each agency had its own unique PCCP fidelity trajectory, the qualitative data illustrated both similarities and differences across clinics. All experienced barriers related to implementing person-centered care planning due to their EHR but their responses to these challenges differed according to the developmental stage of their EHR and their ability to make changes (see Table 1).

**Table 1:** EHR Development Stage, Challenge and Resolution by Clinic

EHR Development Stage	Challenge	Resolution
No Change	Limited ability to individualize service plan	Able to make necessary changes to EHR
Changing	Technical problems	Reverted to original EHR format
Changing	Limited ability to individualize service plan and locked into EHR format	Developed workarounds which were time consuming
Planning Change	Technical problems and locked into EHR format	Unable to resolve challenges
Planning Change	Limited ability to individualize service plan and locked into EHR format	Developed workarounds which were time consuming

The table summarizes the qualitative findings by EHR development stage, challenge, and resolution by clinic

#### Planning EHR change

Both clinics that were planning major changes to their EHR experienced being “locked in” to a system during the PCCP implementation period. The inability to customize their records was due to sharing a platform which limited their ability to change the record to meet their clinical needs. One of the clinics was part of a large integrated health system with a uniform EHR and the other clinic shared a software platform with other clinics. The latter experienced technical problems in terms of the usability of the service plan template. A supervisor described her frustration with difficulties entering information,

*And I, I basically almost started to cry one day… I just got to the point where I just was like, “I can’t do this. This is just adding so much work to my day that I cannot use this form, you know, the way that they designed it” (Site 1, Supervisor).*

Both clinics experienced difficulty personalizing information due to drop down menus giving them uniform options rather than the ability to individualize goals and objectives. One provider described the problem, acknowledging the benefits of uniformity but also showing how this can conflict with the spirit of person-centered care,

*Our system allows us to just click objectives, interventions, and they’re not, they’re all measureable and they’re great, but that piece of it, but I—but they’re not good treatment planning, because they are something that can be achieved quickly, ...it was hard to allow people to be creative… (Site 2, Supervisor).*

When discussing the problem with this clinic’s IT department, the technicians kept suggesting solutions where providers could just “click” options, which providers worried would lead to “cookie cutter” plans. They finally came up with a workaround,

*It can be, but you can pick “other,” and that’s, you can pick “other” or you can put in “in the client’s words.” ... And I just tell staff to don’t ever use a click one, like, just put in your own. So that’s just, like, a different train of thought (Site 2, Supervisor).*

While this clinic came up with a solution, it was time consuming for providers and therefore, a potential disincentive to practicing PCCP. The other clinic struggled to resolve the incompatibility of PCCP with their EHR. It was a constant concern voiced in their technical assistance calls but they were unable to make headway in amending their service plan. Both clinics used their experiences with implementing PCCP to inform the design of the next iteration of their electronic health record.

#### EHR change

Two clinics were in the midst of a major change to their EHR while they were being trained in PCCP. This change disrupted the implementation of PCCP creating technical glitches and barriers to the delivery of person-centered care. For one clinic it led to a false start as they attempted to move towards having a shared service plan across all programs. Providers could see the advantages of greater continuity of care by all working from the same set of goals, but the reality proved unwieldy,

*It was implemented in the middle of a big firestorm with us trying to get onboard with the new Echo system [ph] and dealing with the stress that it created interpersonally between coworkers. And being frustrated, again, trying to call clinicians who were in session, you know, and maybe didn’t get back to you till the next day, so then you had to tell your client to leave because they couldn’t get the signature and then the dates didn’t match up. It was insanity (Site 3, Supervisor).*

Many of the technical assistance calls were concerned with the chaos generated by the shared service plan and trying to figure out a workable template. In the end, the technical glitches for this agency proved insurmountable and they gave up using a shared plan, which frustrated providers as there had been high expectations and investment in the change. For the second clinic, the major change implemented concurrent to the PCCP training specifically undermined the practice of person-centered care. As they made the EHR more uniform, it became a barrier to PCCP,

*One of the barriers with our clinic too when we did our recovery plan was we’d just implemented the new electronic system, so, um, [Supervisor’s Name] has been doing kind of group supervision on how to personalize and edit that because we’re just picking from dropdowns in the electronic system. So to implement the person-centered in that, we have to go in and edit the, um, the like predetermined goals and interventions that the system prompts (Site 4, Direct Care Provider).*

Like other clinics, they figured out a workaround by editing the system prompts, but this created an additional step. In their technical assistance calls, the providers described their new EHR system as too “*medical model*” in that it both reflected and promoted a problem-based approach, with the service plan fields flowing directly from the diagnosis leading to a uniformity in both problems and solutions. By orienting the service plan around the diagnosis and forcing predetermined solutions on providers, the system failed to reflect key principles of the recovery model, which is to understand the uniqueness of each individual and define life goals that are broader than addressing pathology. As with the other clinic, much of the technical assistance time was taken up with problem solving around the EHR format. This included discussions about how to negotiate with the IT workers with requests for free text modifications while maintaining the efficiency of the system.

#### No EHR change

There was one agency who had a fully operational electronic health record that was well established and were not planning any changes. During the training this agency did experience some problems with the incompatibility of their EHR with person-centered care planning with respect to their ability to personalize goals. As one leader described, they were able to address this early on in their training,

*Well we were talking about to our vendor about umm about making some changes and we were able to make some changes, one during the course of the training…that actually helped somewhat… So, actually that’s another step we took to implement Person -Centered Care Planning. So we did make some changes in our EHR that will facilitate a little bit more (Site 5, Leader).*

The clinic was able to work with their vendor and successfully customize their EHR to align with PCCP. As the leader describes, the needs of the intervention helped them articulate changes that they had already been considering. Once they had revised the service plan on the EHR, the clinic then held a training with the vendor present for all their providers on how to use the new service plan template. After this was resolved, the EHR was a much less prominent factor in the implementation of PCCP than for the other clinics and the technical assistance calls focused on other aspects of the implementation.

### Quantitative findings

**Fig. 1:**
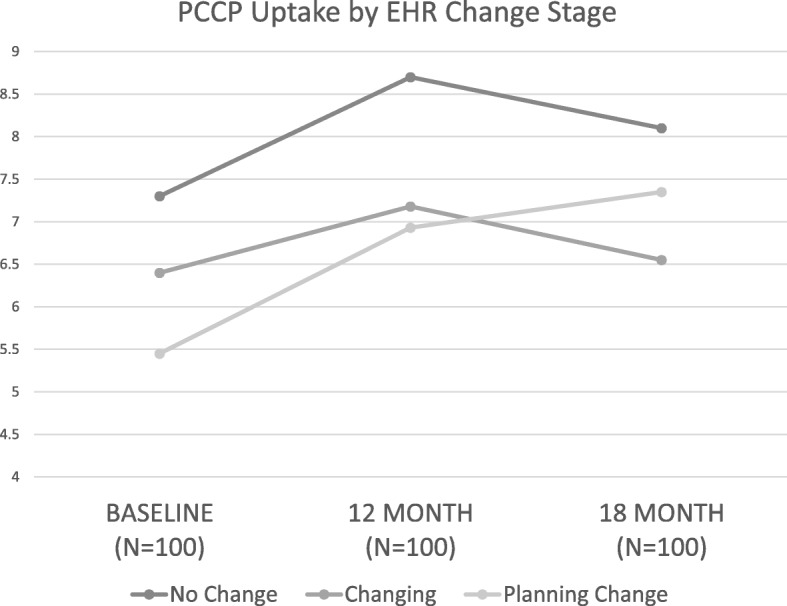
PCCP Fidelity by EHR Development Stage. The figure shows PCCP fidelity score (range 1–13) at baseline, 12 months and 18 months by three EHR development stages at baseline, 12 month and 18 months

Longitudinal analyses demonstrated different trajectories in PCCP fidelity for clinics by EHR stage of change (see Fig. 1). While all clinics demonstrated positive improvement in overall fidelity from baseline to 18 months, there was variation in their baseline, 12 month and 18-month PCCP fidelity by developmental stage. The site with no change in its EHR had higher fidelity at baseline and reached higher levels at 12 month and baseline. Clinics changing their EHR (*N* = 2) started at a higher level of PCCP fidelity but at the 18-month point had the lowest level of PCCP fidelity. Clinics planning a change in their EHR (*N* = 2) started with the lowest levels but increasingly improved their fidelity to end up with a higher level of fidelity compared to clinics that were changing. Significant differences in PCCP fidelity were found according to EHR development stages for baseline (F = 4.5, *p* < .05), 12 month (F = 3.8, <.05) and approaching significance for 18 month (F = 2.9, *p* = .06).

## Discussion

The study findings explored the role of the EHR in the implementation of a new practice initiative aimed at promoting person-centered care in behavioral health settings. When comparing stage of EHR and PCCP fidelity, the clinic with the most established and high functioning EHR demonstrated the highest level of fidelity both at the baseline and 18 months. Clinics differed significantly in their PCCP fidelity according to their development stages suggesting it to be a salient implementation factor. The qualitative findings provided insight into each clinic in terms of their difficulties related to individualizing the record and their capacity to address these difficulties. A fundamental aspect of the PCCP intervention is to have service planning flow out of personalized goals that were meaningful to the service user and be able to express these goals and their objectives using the service user’s own words. Drop down boxes and predetermined goals that created the logic and workflow of these clinics’ EHRs could not adequately reflect the uniquely varied nature of goals determined by PCCP. This limitation made it impossible for providers to practice according to the recovery model, which acknowledges the uniqueness of each person’s recovery trajectory and that goals must be personal and meaningful. Problem solving on how to address these barriers consumed a considerable part of the technical assistance calls for most clinics taking away time spent reflecting on the more clinical aspects of the practice.

Some of the problems that clinics encountered were more generic to launching a new practice initiative while simultaneously making changes to the electronic health record. The clinics that were actively changing their EHRs experienced technical difficulties and general disruption due to their EHR being in flux. The clinics that were planning changes to the EHR also faced technical problems, which in part were driving the need to upgrade their systems. The findings reflected the iterative nature of embedding an EHR system into a clinic, which can create challenges for the day-to-day clinical practice. This echoes prior findings which demonstrate that while there can be benefits in terms of efficiency and coordination care are clear, the reality of shifting to EHR systems can often undermine these goals leading to mixed outcomes [3, 27].

Solutions were found either in the form of customization, or if that was not possible with the clinic’s software by devising “workarounds”. The clinic with a well-established EHR had the flexibility and support to make the necessary structural changes relatively easily. Similarly, Goh and colleagues [5] found that a key part of a successful EHR transition was having the capacity to modify a HIT system quickly when it is found to be incompatible with a routine practice, or in this case a new practice. The clinics which were not able to respond quickly to these barriers were the clinics that had less control over the formatting of their record due to being part of a larger system or sharing EHR infrastructure with other clinics. The only option for the clinics that could not alter their record was to develop “workarounds”, which in the case of individualizing the record meant “picking other” to have the option to enter free text or editing pre-determined goals to reflect individual preferences. These workarounds while enabling a more person-centered approach were time consuming and undermined the efficiency and uniformity of the data entry. Workarounds are a common compromise in using electronic health records and often considered to be essential by providers [28]. However, others have argued they ultimately reflect “mismatches between the capabilities of the existing HIT system and the clinical practices need to perform” (3, p.S65).

The reason for mismatches with clinical practices is partly rooted in the fact that the EHR does not have the sole purpose of facilitating person-centered care but instead has multiple purposes that often require uniformity for the purposes of aggregation. The increased focus on improving population level outcomes and the potential for the EHR to support this goal is an incentive for constructing EHRs in ways that promote aggregation [29]. While person-centered care and population level care both represent key elements of the Triple Aim – they require potentially contrary EHR functionality. Another predominant use of the EHR is to inform billing, which requires establishing “medical necessity criteria” for multiple clients across different programs in a way that is feasible for the agency. This inevitably leads to uniformity in the ways the problem and the solutions are conceptualized, which in turn drives more medical model approaches to care. As several of our agencies experienced, the logic of the record flows specifically from the diagnostic problem rather than personalized goals. This study demonstrated that despite electronic health records being a key strategy in improving quality through coordination of care and efficiency, the EHR can also be a barrier to the delivery of person-centered care.

### Limitations

There are several limitations to this study which indicate the need for further exploration of this subject area. First, there are other factors not accounted for in our quantitative analysis that could have led to variation in fidelity to PCCP, though qualitative data supports the finding that the EHR had a substantial influence on the implementation process. Because of this, while it can be concluded that EHRs played a significant role in the adoption of this new practice, these findings cannot quantify the magnitude of this influence, or speak to the isolated role of EHRs relative to other factors that may have also impacted implementation. Furthermore, only one site experienced no change in their EHR system, minimizing variation within this category, and limiting the differential impact EHR change stage had on PCCP fidelity.

## Conclusion

This study provided insight into the relationship between technology and person-centered care in behavioral health care settings. Ongoing provider concerns and resistance to integrating EHRs into clinical tasks speak to an underlying concern that somehow EHRs are infringing on the essentially human part of the work, which is a fundamental part of person-centered care. Health care providers increasingly feel that rather than EHRs facilitating practice the technology is instead driving practice in ways that undermine interpersonal interaction [30]. In behavioral health specifically, the fact that providers spend more time in front of the computer and that care can become more uniform and less personalized due to EHR formats can prove a barrier to recovery-oriented practice. The result being that policy efforts designed to promote EHR adoption and care coordination may have the unintended consequence of diminishing person-centered care. As EHRs are increasingly being used in behavioral health settings, ensuring that systems are also responsive to and reflective of the unique needs of the service user is essential to maintaining high quality services.

## Data Availability

The de-identified datasets used and/or analyzed during the current study are available from the corresponding author upon reasonable request.

## Abbreviations

EHR: Electronic health record
HIT: Health information technology
PCCP: Person-centered care planning
PCCP-AM: Person-centered Care Planning Assessment Measure
TA: Technical assistance
